# Deep learning techniques for detecting and recognizing face masks: A survey

**DOI:** 10.3389/fpubh.2022.955332

**Published:** 2022-09-26

**Authors:** Rahaf Alturki, Maali Alharbi, Ftoon AlAnzi, Saleh Albahli

**Affiliations:** ^1^Department of Information Technology, College of Computer, Qassim University, Buraydah, Saudi Arabia; ^2^Department of Computer Science, Kent State University, Kent, OH, United States

**Keywords:** face mask, public health, crowd monitoring, transfer learning, convolutional neural network

## Abstract

The year 2020 brought many changes to the lives of people all over the world with the outbreak of COVID-19; we saw lockdowns for months and deaths of many individuals, which set the world economy back miles. As research was conducted to create vaccines and cures that would eradicate the virus, precautionary measures were imposed on people to help reduce the spread the disease. These measures included washing of hands, appropriate distancing in social gatherings and wearing of masks to cover the face and nose. But due to human error, most people failed to adhere to this face mask rule and this could be monitored using artificial intelligence. In this work, we carried out a survey on Masked Face Recognition (MFR) and Occluded Face Recognition (OFR) deep learning techniques used to detect whether a face mask was being worn. The major problem faced by these models is that people often wear face masks incorrectly, either not covering the nose or mouth, which is equivalent to not wearing it at all. The deep learning algorithms detected the covered features on the face to ensure that the correct parts of the face were covered and had amazingly effective results.

## Introduction

The COVID-19 virus took the world by surprise and it still looms large as a threat. According to WHO estimates released on 6 December 2021, there were 265,194,191 confirmed cases and 5,254,116 deaths in 200 countries (1). There is an urgent need for a solution to reduce the virus's spread, which can be achieved by following WHO rules of keeping social distance and wearing face masks.

COVID-19 is a virus that originated from Wuhan, China and it causes illnesses similar to the common cold, severe acute respiratory syndrome (SARS) and Middle East Respiratory Syndrome (MERS). In March 2020, it was declared a pandemic by the World Health Organization (WHO). Its symptoms include fever, cough, shortness of breath, running nose, sore throat and tiredness, which begin to show after 2 to 14 days from exposure. The most deadly aspect of the virus is that some people can have it without showing any symptoms early on but are still able to transmit it.

Many public service providers require customers to wear masks and maintain a safe distance while using their service. However, some people, consciously or unconsciously, violate these precautionary and preventive measures, and this makes it difficult to monitor the effectiveness of these guidelines on a large scale (nationally or globally). For an airborne disease, abiding by these preventive measures goes a long way in effectively controlling the spread of the virus, but due to human error, most people still fail to adhere to them. Even though face masks do not on their own stop the spread of the virus, when combined with other preventive measures, such as washing of hands and social distancing, face masks play a major role in stopping the spread of the disease. The WHO say masks should be used as part of a comprehensive strategy of measures to suppress transmission and save lives and they urge people to make wearing a face mask a normal part of being around other people (2).

For that reason, monitoring methods are being sought by governments, companies, and many public service providers to keep track of people who do not follow the preventive measures. This is a hard task as there has to be constant monitoring, because many people might wear a face mask and later on decide to remove it or wear it the wrong way, and this needs to be monitored. This monitoring needs to be in real time and should be optimally performed, because removal of the mask, even for just a second, could be catastrophic.

Deep learning, which is a type of machine learning technology that has shown success in several fields, including image recognition, anomaly detection, pattern recognition, and natural language processing, could be used in combination with computer vision techniques to create a robust model that is able to serve such a purpose. Deep learning has played a major role in progress toward using artificial intelligence in detecting and diagnosing medical conditions, mostly by employing computer vision.

Computer vision is a branch of artificial intelligence (AI) that enables computers and systems to extract useful information from digital photos, videos, and other visual inputs, as well as conduct actions or make recommendations based on that data. Computer vision works by identifying certain features on the images used for training: then it associates these features to determine which category a particular image belongs to, either wearing or not wearing a face mask. After training, the model can effectively categorize input images using the features to determine whether or not a person has a face mask on. A huge amount of data is required for computer vision. It repeats the data analysis process until it recognizes distinctions and, eventually, images.

The main objective of this survey is to discover ways of detecting and identifying people who fail to comply with the mandated rule of wearing face masks in public areas. It will use frames of images to extract already identified features to determine if a person has a face mask on, and if they do, whether they are wearing it correctly.

The model, which is based on deep learning, will start by reading the input frame, then processing it through the object detector until it finally delivers the desired output. The main contributions of this survey are highlighted as follows:

1. To study state-of-the art research that has been conducted into face recognition, masked face detection (both properly and not properly worn).
2. To effectively review available datasets available relating to face masks and COVID-19 and how they were gathered.
3. Review Masked Face Recognition (MFR) and Occluded Face Recognition (OFR) deep learning algorithms and study how well they detect correct usage for face mask.

### Motivation of the study

The world recorded thousands of COVID-19 deaths within a span of a few months, leading to a global state of emergency and disrupting the world economy. When the World Health Organization (WHO) announced preventive measures, the rate of spread of the virus drastically reduced; however, due to human nature, these measures are not always strictly followed and this tends to sabotage the efforts of public health workers. This indicated the need for this research as it aims to find an effective way of detecting and identifying people who do not comply with the face mask rule, so as to ultimately reduce the spread of the disease.

### Challenges and gaps

**Figure 1:**
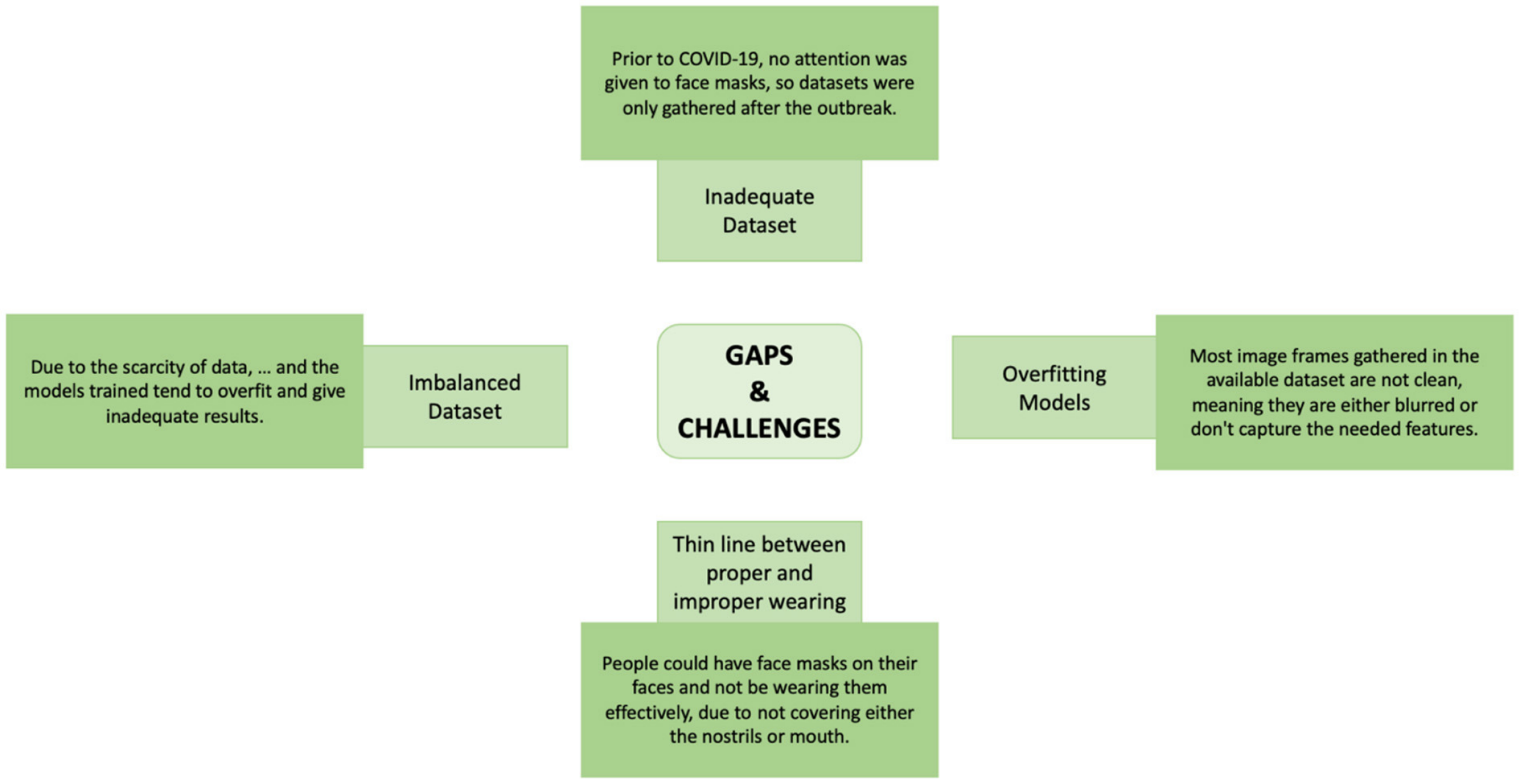
Gaps and challenges.

Figure 1 shows the possible research gaps and challenges likely to be faced with deep learning models for automatic face mask detection.

#### Research questions

The following questions are answered in this research:

1. Could artificial systems effectively detect masked and unmasked faces automatically?
2. Could the systems detect if masked faces have the face masks properly worn? If so, how effectively can they determine this?

### Search methodology and taxonomy level

Face mask detection is the process of identifying whether an individual is wearing a face mask or not in a picture or moving frame. In public places, the wearing of a mask is considered an important safeguard against the spread of COVID-19; therefore, monitoring methods are urgently needed and are being sought by governments, companies, and many public service providers.

**Figure 2:**
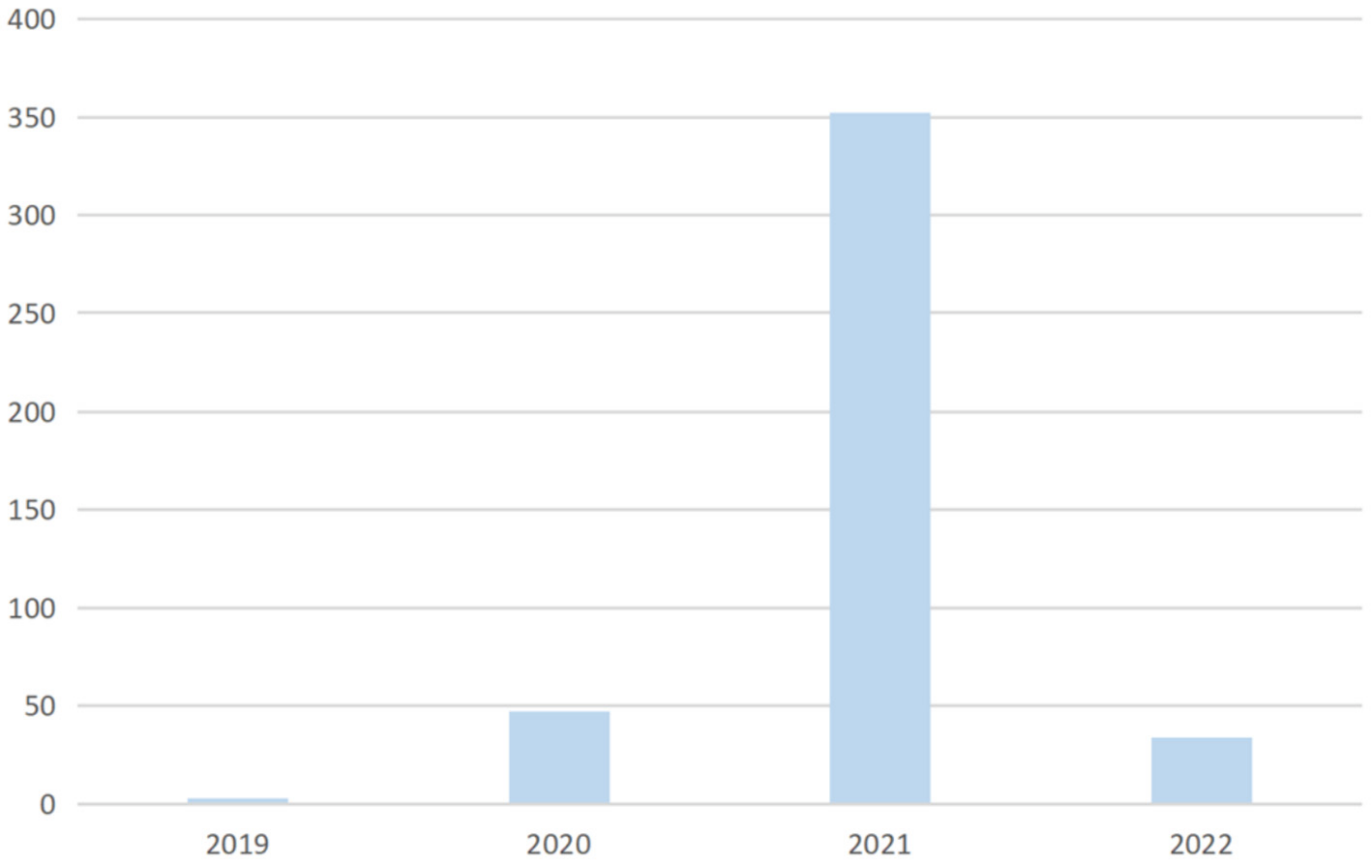
Demonstration of research efforts on face mask detection from 2019 to 2022.

A search query is performed to track efforts on face mask detection over the years. Figure 2 clearly indicates that the increase of research related to face mask detection goes hand-in-hand with the spread and increase of corona virus cases, since the virus is a major reason for implementing face mask detection techniques.

#### Taxonomy level

**Figure 3:**
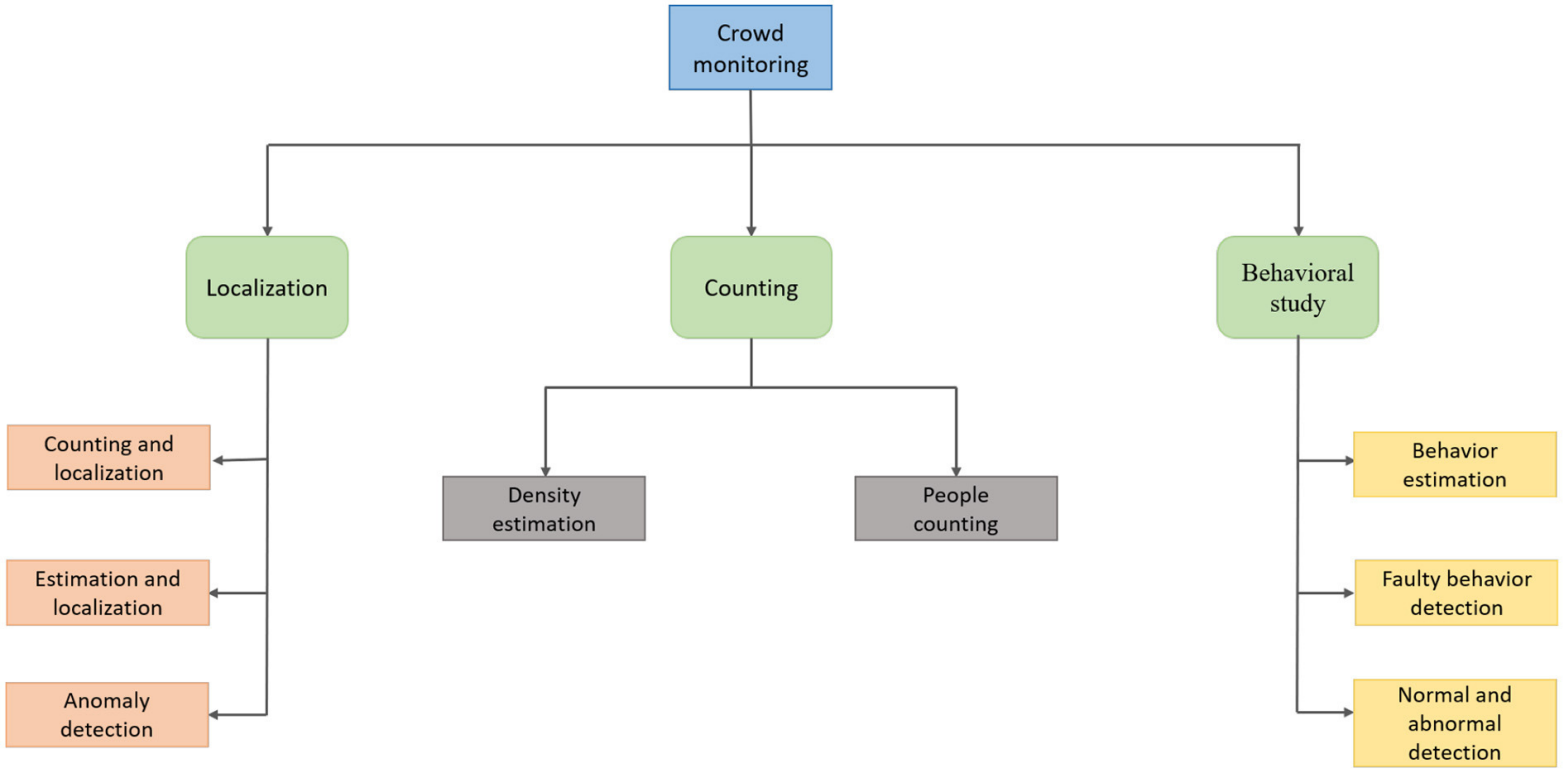
Pipeline for crowd monitoring.

Crowd monitoring comes down to three things: localization, counting and behavioral study. These are the pipelines followed by the techniques in order to effectively and efficiently segregate those in breach of the face mask rule. Figure 3 shows the pipeline for crowd monitoring; it shows how localization of the faces is carried out, how crowd density is measured through counting and how the behavior of the crowd is studied.

## Related studies

Due to COVID-19 being a relatively new virus, surveys conducted with regards to face masks are very limited. Nowrin et al. (3) comprehensively reviewed the studies done on face masks in the context of COVID-19. They also discussed the progress of object detection algorithms in solving such problems over the past few decades. Mbunge et al. (4) reviewed AI models used for face mask detection, as well as the available datasets for this task. After reviewing the datasets, they concluded that most of them were artificially created due to lack of datasets, and therefore wouldn't perform well in a real-life situation. Batagelj et al. (5) focused on the problem of detecting only face masks properly worn and discusses the feasibility of existing models that detect face masks, as most of them detect the presence of the mask regardless of the way it is worn. Wang et al. (6) worked on analyzing 13 open/masked face detection datasets, discussing their benchmark results and limitations.

### Face recognition

Face recognition, which is a computer-based biometric information technique that is used to identify or verify an individual from a picture or a video frame (7), is a fundamental concept in deep learning. Face recognition is one of the most researched topics in the field of computer vision, with hundreds to thousands of studies undertaken. Compared to other biometric-based methods, such as fingerprints and iris, human faces are a much better way to recognize someone's identity. Thus, facial recognition features have been used extensively in various applications, including forensics, security checks, and many other uses. Face recognition is a long-standing concern in computer vision, with a rich history; hence, there are diverse approaches to it. Yet, several factors negatively affect the performance of Facial Recognition (FR) algorithms, including the presence of occlusions and variations in lighting.

Research done by Adinj et al. (8) stated that the average misclassification and failure rate was above 50% and never less than 20%, even with the best representation of an image. Research done by K Wickrama Arachchilage and Izquierdo (9), presented the origin and evolution and a comparative analysis of 18 face recognition systems. Through this, the survey's aim was to provide informational recommendations in order to model future research. In doing so, the paper analyzed the performance of the systems, based on benchmark results reported on three benchmarks which address different aspects of face recognition and an experimental study. The research in Zhou and Xiao (10) categorized 3D face recognition systems into pose-invariant recognition, expression-invariant recognition and occlusion- invariant recognition. It also provided an overview of publicly available 3D face databases. Also, 3D face recognition technology has been applied in many fields, such as access control and automatic driving.

Although face recognition research laid the groundwork for research into masked face detection, it doesn't get the job done because detection deals with identifying features which confirm it is a face, but the mask covers half or even more than half of the face. Hence, this is taken a step further to research models which could detect covered faces.

### Masked face recognition

For the task of Masked Face Recognition, the traditional methods of FR do a poor job in handling complex and unconventional faces, which raises the demand for more effective masked-face recognition systems. Unlike FR, Masked Face Recognition (MFR) is mostly COVID-19 related, and hence the amount of study on this topic has dramatically increased with the outbreak of the virus. Work on MFR is basically an extension of the work on FR. In order to be able to address the issue of identifying faces with masks, the scientific community has been developing and improving the existing methods for face recognition and occluded face recognition.

With the aim of recognizing masked faces, a recent study by Hariri (11) proposed a deep learning-based method that works by removing occlusion. The method works by removing the mask part from the face, this is considered a type of face occlusion. After that, Convolutional Neural Network (CNN) models are used to extract the features from the remaining part of the face (forehead and eyes). Three CNN models were used, namely AlexNet, ResNet-50, and VGG-16. The Bag-of-features (BoF) paradigm is then employed in the last convolutional layer's feature maps for the sake of quantizing them. This BoF paradigm does a job similar to that of Bag of Words in Natural Language Processing; it is used to find the image in the database which is closest to a query image. Lastly, for the classification process, Multilayer Perceptron (MLP) is utilized. The evaluation results on the dataset used presented high performance when compared to other state-of-the-art techniques.

Anwar et al. (12) proposed an open-source tool named MaskTheFace that synthetically produces a collection of faces covered with masks. To professionally mask faces, even when they're titled, it detects six key features of the face that are essential for applying the mask. Then, the suitable mask template is chosen from the mask library and fitted to the face based on the six features. To address the challenge of identifying faces with a mask on, which is critical for security systems, Mandal et al. (13) developed a model that is capable of correctly identifying individuals with a face mask. The architecture is based on ResNet-50. For training, supervised domain adaptation was considered. Unmasked faces were marked as the source domain (S), and masked faces as the target domain (T). Then, two conditions were used to train and test the models. The first model was trained using only S and the performance was tested using T. The second model was trained on the entirety of S and some parts of T and tested on the remaining parts of T.

In COVID-19, wearing a mask has become the norm, which makes the task of face recognition and authentication more challenging. Four major studies (14–17) were published to address the effect of face masks on face recognition systems. Two studies were published by The National Institute of Standards and Technology (NIST), which focused on analyzing the performance of “pre-pandemic” face recognition algorithms (14) and “post-pandemic” face recognition algorithms (15) on faces covered by protective face masks. Both studies concluded that masked faces affect the performance of face recognition negatively, resulting in lower accuracy in comparison to unmasked faces. It is noteworthy that NIST studies used artificially generated masks for evaluation, assuming that they had the same effect as real masks, which is debatable.

On the other hand, the study published by The Department of Homeland Security (16) has a similar evaluation, except that they used real masks. The study again found that wearing a face mask has a negative impact on face recognition systems. In order to have the most realistic experiment, the study conducted by Damer et al. (17) worked on collecting their own dataset that covers different challenging conditions, such as noise, illumination, etc. The dataset was then tested on three algorithms. Two academic approaches, namely SphereFace (18) and ArcFace (19) and a Neurotechnology commercial off-the-shelf (COTS) system (20). The study also pointed out a significant decline in the verification performance of academic face recognition systems.

### Face mask detection

Fan et al. (21) developed a new single-stage face mask detector called RetinaFace mask. They started by creating a new face mask detection dataset, named MAFA-FMD. The face mask images in the MAFA-FMD dataset are labeled as “mask” only if they are proper masks that can actually provide prevention against the transmission of any airborne viruses, and hence it provides a more accurate classification of a mask-wearing state. The object detector utilized ResNet50's network for feature extraction and FPN for high-level semantic information. The researchers also developed a light-weight version of the face mask detector using the backbone of MobileNetV1. The proposed method achieved phenomenal results on the publicly available face mask dataset as well as the MAFA-FMD dataset. The mean Average Precision (mAP) of the face mask detector was higher by 4% when using the MAFA-FMD dataset in comparison to the public dataset AIZOO (22).

Dhanushkodi et al. (23) proposed a system that can detect whether or not a person is wearing a face mask, as well as send an alert to the administrator if a person is found without one. The system might also keep track of people's social distance and detect if somebody is failing to maintain it. Using a Faster R-CNN model in a convolutional neural network, this study suggests a deep CNN framework for detecting and labeling face mask and social distancing observance. The Faster R-CNN model was created by adding some extra layers to the CNN of the Fast RCNN architecture, which conducts regression and classification simultaneously. Convolutional layers collect information from images and generate region proposals from the convolutional feature map with several area proposals in order to detect a specific object.

The goal of the work done by Loey et al. (24) is to develop a model that is able to detect the wearing of a face mask in real life images. Using a face mask to cover the mouth and nose area helps to reduce the transmission of COVID-19. The developed model is composed of two parts. The first part is concerned with feature extraction. The ResNet-50 model was employed for this task. The second part is concerned with the detection of face masks. Yolo2 was utilized for this. For this study, two face mask datasets were combined into a single dataset, namely MMD dataset and FMD dataset, both of which are publicly available on Kaggle. Both Adam and SGD optimizers were used, though the Adam optimizer achieved better results, with 81% mean Average Precision (mAP) in comparison to the SGD optimizer, which scored only 61%.

Several factors affect the ability of models to detect face masks; these include light, occlusion, and multi-object detection. To effectively handle these challenges, the study by Su et al. (25) proposed a fusion of transfer learning and the Efficient-Yolov3 face mask-wearing detection algorithm. To increase the speed and accuracy of model detection, the approach uses EfficientNet as the backbone network for feature extraction and CIOU as the loss function. Simultaneously, transfer learning helps improve the model's training speed and generalization capabilities. A face mask classification dataset was also constructed, which divides masks into qualified masks (N95 masks, disposable medical masks) and unqualified masks (cloth masks, sponge masks, scarves, etc.). A mask classification technique based on fusion transfer learning and MobileNet is proposed. The algorithm developed in the research has been experimentally proven to effectively distinguish the types of masks, producing precise classifications with an accuracy of 97.84 percent. Table 1 shows a brief summary of the studies carried out on Face Mask Detection using deep learning algorithms.

**Table 1:** Summary of face mask detection works.

**Reference**	**Methodology**	**Dataset**	**Performance**
AIZOOTech (22)	RetinaFace mask	MAFA-FMD	High accuracy
Dhanushkodi et al. (23)	R-CNN	-	High accuracy
Loey et al. (24)	ResNet-50 Yolo2	MMD and FMD dataset	81% mAP
Su et al. (25)	Fusion transfer learning Effcient-Yolov3 EffcientNet	-	97.84% Accuracy

### Social distance tracking

Social distancing is a proven strategy for effectively slowing the spread of the virus, and is defined as keeping a minimum of 1 meter between people so that they do not come into contact with one another (26). In a study conducted by Greenstone and Vishan (27), moderate social distance implementation is shown to be economically advantageous, as it reduces fatalities. Hence, irrespective of the fact that COVID-19 is unlikely to be eliminated soon, a computerized system that allows the monitoring and analysis of social distancing behaviors would be remarkably beneficial to our society. Researchers use CCTV videos, along with computer vision, and deep learning-based algorithms, to develop effective solutions for social distance measurement.

Yang et al. (28) proposed an AI real-time system that uses monocular-cameras to detect and calculate the distance between individuals without the data being recorded. If the system detects any social distance violations, it will deliver a visual-audio alert to notify the crowd. The system was evaluated using YOLOv4 and R-CNN, both of which are types of deep-CNN-based object detectors. YOLOv4 and R-CNN achieved mean average precision (mAP) of 41.2–43.5 and 42.1–42.7 respectively.

Two models were employed in the suggested framework of Madane and Dnyanoba (29). The first is EfficientDet, which is created by combining an EfficientNet backbone and weighted BiFPN. The DEtection Transformer (DETR) with resnet50 backbone is the second model. It works by predicting all objects at the same time, using a set-based loss that uses bipartite matching to provide unique predictions. Basic positional augmentations, such as flipping, resizing, and rotating, were employed as varied lighting conditions, video quality, and occlusions can affect the detector's accuracy. The camera angle for the input view is turned into an overhead view, which provides a better representation of reality in comparison with the actual input data, so the estimation of the distance between individuals becomes more accurate. Two public datasets, Oxford Town Center (OTC) and PETS (people tracking) were used to evaluate the models. The study concluded that DETR surpassed both variants of EfficientDet (D0, D5 backbones) with an mAP score of 94.23, while D0, D1 scored mAPs of 83.89 and 88.91, respectively.

Ahmed et al. (30) used an overhead perspective to track social distance. To recognize individuals in video sequences, the system employs the YOLOv3 object recognition paradigm. For the purpose of improving the model's accuracy, researchers considered using transfer learning approaches. Transfer learning allows the system to be further taught without losing any of the previous model's useful data. In addition, a layer trained on an overhead dataset is added to the existing architecture. As a result, the model uses both pre-trained and newly-trained information, resulting in enhanced detection and delivery times. To identify humans, the detection model makes use of bounding box information. The Euclidean distance is used to compute the distance between the centroids of the observed bounding boxes. In order to detect violations of social distancing between people, a specified threshold is employed to verify whether the distance between the centroids of any two bounding boxes is smaller than the decided number of pixels.

For social distance monitoring in the work done by Suryadi et al. (31), three models were employed for classification: namely, YOLOv3; YOLOv3 -Tiny, which is a lightweight version of the first model; and MobileNetSSD. which essentially combines Single Shot MultiBox Detector (SSD); and MobileNet for feature extraction or prediction. The three models were trained on the Microsoft Common Objects in Context (MS COCO) dataset and then examined on recorded CCTV videos of pedestrians in an Oxford location. The results of this study shows that YOLOv3 outperformed both YOLOv3-tiny and MobileNetSSD regarding detection accuracy, but at the expense of a heavy, extremely time-consuming computational process.

For the purpose of detecting and tracking people and estimating the distances between them, Rezaei and Azarmi (32) developed a robust model named DeepSOCIAL, which is based upon YOLOv4 with CSPDarknet53 as a backbone. These two together combine speed and accuracy for multiple classes of object detection, considering that the system is intended to be used in all outdoor areas, using only basic surveillance cameras. Google Open Image datasets, along with the MS COCO dataset, were used to train the model. To take advantage of the MS COCO's pre-trained models, researchers decided to use transfer learning, followed by fine-tuning of the YOLO-based model. Distance calculation might not be so accurate due to the perspective effect problem. To overcome this issue, DeepSOCIAL adopted the inverse perspective mapping (IPM) technique. When the system was evaluated, it reached a real-time speed of 24.1 frames per second and a mean average precision of 99.8% under difficult conditions, such as partial visibility, occlusion, and various lighting conditions.

To computerize the process of social distance monitoring, Punn et al. (33) developed a real-time deep learning-based framework. The study focused on the scope of object detection and tracking. A bounding box surrounds each individual for detection in real-time. Finding that magical balance between speed and accuracy is a keystone in computer vision real-time systems. For that purpose, YOLOv3 and Deepsort were utilized as object detection and tracking techniques. It is crucial for an object detector to be able to handle difficult and challenging situations such as detecting from different viewpoints, moving cameras and poor lighting conditions. Luckily, Deepsort which is a deep learning object tracking algorithm can take care of all that. The results delivered by this study using Yolov3 with Deepsort were phenomenal, as it scored a balanced FPS and mAP of 23 and 84.6, respectively. Table 2 shows a detailed comparison of the social distancing monitoring deep learning architectures that were reviewed in this paper.

**Table 2:** Comparison of social distancing detection approaches.

**Specifications**						**Performance**		
**Reference**	**Detector**	**Perspective**	**Dataset**	**Transfer** **learning**	**Real-time system**	**mAP**	**Inference time**	**FPS**
Greenstone and Vishan (27)	YOLOv4	Transformed into overhead	Old Town Center (31)	No	Yes	42.1–43.5%	0.048	-
			New York Grand Central Station (32)				0.05	-
			The Mall Dataset (33)				0.05	-
	Faster R-CNN	Transformed into overhead	Old Town Center (31)	No	Yes	42.1–42.7%	0.145	-
			New York Grand Central Station (32)				0.116	-
			The Mall Dataset (33)				0.108	-
Yang et al. (28)	EfficientDet-D0	Transformed into overhead	Pets (34)	Yes	Yes	83.98%	0.0541	19
			Oxford Town Center (31)				0.0593	
	EfficientDet-D5	Transformed into overhead	Pets (34)	Yes	Yes	88.91%	0.0831	12
			Oxford Town Center (31)				0.1040	
	Deter	Transformed into overhead	Pets (34)	Yes	Yes	88.91%	0.0478	21
			Oxford Town Center (31)				0.5003	
Madane and Dnyanoba (29)	YOLOv3	Overhead	MS COCO Dataset (35)	No	No	84%	-	-
	YOLOv3		MS COCO Dataset (35)	Yes *Additional Training is done using an overhead dataset	No	86%	-	-
Ahmed et al. (30)	YOLOv3	Frontal	MS COCO Dataset (35)	No	No	-	-	12.98
	YOLOv3-Tiny					-	-	37.35
	MobileNetSSD					-	-	8.44
Suryadi et al. (31)	DeepSOCIAL (YOLOv4-CSP Darknet53)	Transformed into overhead	MS COCO Dataset (35), Google Open Image Dataset (36)	Yes	Yes	99.8%	-	21.4
Rezaei and Azarmi (32)	YOLOv3	Frontal	Google Open Image Dataset (36)	No	Yes	84.6%	-	23

### Datasets

This section provides a detailed discussion of some of the most commonly used datasets in face mask detection algorithms, looking at their content, size, and existing experimental results. One of the most important criteria for developing deep learning algorithms is a large number of training sets. Due to the nature of model training, large datasets are necessary to perform mask detection tasks if deep learning algorithms are to be used. Although a large number of face datasets are available for face detection and recognition, the number of faces associated with masks is very low. However, the number of masked face datasets rose as soon as mask detection methods received attention in the post-COVID-19 world.

#### Description of datasets

First, we present an earlier dataset for masked face detection. In Ge et al. (34), a large dataset called MAFA was proposed. MAFA contains 30,811 images gathered from the internet, with 35,806 images of masked faces. The dataset is labeled with six attributes: location of the face, location of eyes, location of mask, face orientation, occlusion degree, and mask type. This dataset is more suitable than previous efforts for occluded face detection and it solved the problem of an imbalanced dataset, because it includes a wide range of mask types, incorporating different colors, types, orientation and how different people wear them.

Wang et al. (35) created the Masked Face Detection Dataset (MFDD), a dataset with only one class: it contained 4,342 images of masked faces, with no unmasked faces. It is suitable for training models to determine if a person is wearing a face mask, but it lacks annotation.

Cabani et al. (36) developed MaskedFace-Net with the purpose of generating simulated correct/incorrect masked faces called “MaskedFaceNet Image Dataset (MFNID)”. Candidate face detection, facial landmark detection, mask-to-face mapping, and manual image filtering are the four phases in the framework. The images are divided into two groups: Correct Masked Face Dataset (CMFD) and Incorrect Masked Face Dataset (IMFD). The dataset is very large, with a total number of 137,016 images of both correctly and incorrectly masked faces. It has 67,193 images of correctly masked (49%) faces and 69,823 images of incorrectly masked (51%) faces. For every image, the facial region accounts for a large ratio, making face detection easy. MFNID, on the other hand, only has one type of simulated mask and no annotations.

Jiang et al. (37) presented the PWMFD Dataset (Properly Wearing Masked Face Detection). They gathered 9,205 images from MAFA (34), MFDD (35), and the Internet, among other sources. Although each dataset has its own annotations, the PWMFD dataset provides standard human annotation for three classes: “With mask,” “Without mask,” and “Incorrect mask”. Face regions with nose uncovered are annotated as “Incorrect mask”. There are 7,695 “With mask” faces in the PWMFD dataset, 10,471 “Without mask” faces, and 366 “Incorrect mask” faces.

Eyiokur et al. (38) produced the Unconstrained Face Mask Dataset (UFMD) by aggregating images from the FFHQ, LFW, CelebA, YouTube videos, and the Internet. These publicly available images enable UFMD to be a complex dataset that spans different ethnicity, age range, gender, and location. In UFMD, a large number of head posture variations are taken into account, which helps to improve the robustness of masked face detectors. There are 21,316 images in the UFMD dataset, divided into three categories: 10,618 images with masked faces, 10,698 images without masks, and 500 images with incorrect masks.

Prajnasb (39) proposed the SMFD dataset, which was completely simulated by matching faces to masks. All of the original images were taken from the internet. It has two types of faces with annotations: 690 images with masks and 686 images without masks.

The Kaggle (40) dataset has three types of faces: images of faces without masks, then those that wear them correctly, and those who wear them wrongly. It consists of 853 images in total: 3,232 with masks, 717 without masks, and 123 incorrectly worn.

Wang et al. (41) proposed a Wearing Mask Detection (WMD) dataset that has 7,084 images. The majority of the images were gathered from COVID-19 combat simulations in China, allowing the dataset to be based on real-world circumstances. The dataset has a total of 26,403 images of masked faces: 17,654 for training, 1,936 for validation, and 6,813 for testing. It's worth noting that the test set is split up into three sections based on the difficulty of the detection task and the number of masked faces in each image: DS1, DS2, DS3. In DS1, there are 500 images with only one masked face and relatively larger images. In DS2, there are 500 images, each containing two to four masked faces, making a total of 1,458 masked faces. Each of the 594 images in DS3 contains over five masks, making a total of 4,855 masked faces, and the distance between the face and the camera is substantial (>2m).

In summary, Table 3 shows extensive information about the aforementioned datasets.

**Table 3:** Breakdown of the reviewed available datasets.

**Reference**	**Dataset name**	**Image number**	**Category**	**Masks number**	**Annotation**	**Open**
Ge et al. (34)	MAFA	30,811	Multiple mask types	35,806 masked faces	Yes	Yes
Wang et al. (35)	MFDD	4,342	One	24,771 masked faces	No	Yes
Cabani et al. (36)	MFNID	137,016	Two	67,193 faces with correct masks; 69,823 faces with incorrect masks	No	Yes
Jiang et al. (37)	PWMFD	9,205	Three	10,471 without masks; 7,695 correct masked; 366 incorrect masked	Yes	Yes
Eyiokur et al. (38)	UFMD	21,316	Three	10,698 without masks; 10,618 correct masked; 500 incorrect masked	Yes	Soon Open
Prajnasb (39)	SMFD	1,376	Two	Faces without masks: 686 masked faces:690	Yes	Yes
Kaggle (40)	Kaggle	853	Three	Without a mask: 717 correct masked: 3,232 incorrect masked: 123	Yes	Yes
Wang et al. (41)	WMD	7,804	One	Masked faces: 26,403	Yes	Yes

#### Discussions of datasets

Two aspects will be discussed in relation to these datasets: Image Sources and Class Inequality.

A) Image Sources: The vast majority of datasets were compiled from images found on the Internet. WMD (41), MFDD (35), SMFD (39), and Kaggle (40) used Internet searches to create their images. An advantage of this is that the internet gives a variety of images, which strengthens the dataset gathered. In addition, a larger dataset can be quickly created by combining multiple datasets; researchers should use this method to create their own datasets.
B) Class Inequality: As far as multiple categories of object detection are concerned, the problem of imbalance between classes is clear. In training a model with an imbalanced dataset, erroneous detections are likely. In general, head classes are easier to learn than tail classes. We refer to high ratios of classes as “head classes”, while low ratios of classes are referred to as “tail classes”. Collecting as many images as possible from available datasets can lead to a solution to this problem.

#### Ethical limitations of datasets

A dataset that relates to faces has many privacy issues attached to it because, for one reason or another, people might not want their pictures available on the internet or in a researcher's work. Since the dataset used for most research on masked faced detection uses an already gathered dataset, the onus falls on the owners of the dataset to get the necessary consent from the individuals whose images are used. Some researchers gather their own dataset, in which case, the researchers must obtain consent from the individuals directly to avoid legal harassment.

## Methodology

Deep learning has been employed extensively in battling COVID-19 by using Convolutional Neural Networks (CNN) to classify pictures and videos that may detect or prevent COVID-19. This classification ranges from binary to multi-class classification. However, CNN faces many challenges in achieving accuracy due to the limited dataset available because the quality of the data used for training and testing are the two key factors in building an artificial detection system (42). A CNN is made up of the components below:

1. Convolution layer: This is the main building block of a CNN. It comprises a set of kernels (or filters), parameters of which are to be learned during training. The size of the filters is usually smaller than the actual image and the filters scan the image and create an activation map.
2. Rectified Linear Unit (ReLU): This is an activation layer that helps prevent exponential growth in the computation required to operate the neural network and it sets the negative input value to zero.
3. Maxpooling layer: This is a pooling operation that selects the maximum element from the region of the feature map covered by the filter, thereby extracting the most prominent features from the previous feature map to a new feature map.
4. Batch normalization: This layer re-centers and re-scales input layers so as to normalize them, thereby making training faster and more stable.
5. Fully connected layer: This layer multiplies the input by a weight matric and then adds the bias vector, which gives the probability values for classification into various groups.
6. Loss function: This is a way of evaluating how well a CNN algorithm models the dataset used for training. It does this by applying a soft-max layer to the input data sample.

### Face detection using deep learning

In this section, existing approaches to face mask detection are reviewed, analyzed, and their performance and limitations are discussed. Due to the superior performance of CNN-based algorithms in feature extraction, most face mask detection models utilize CNN-based techniques. CNN-based algorithms outperform other techniques in feature extraction, which is why they are the most used method for face mask detection models. Some studies considered an approach that aggregates classical ML techniques and CNN deep-learning ones, i.e., a hybrid approach. A few studies used solely classical ML, hand-crafted techniques.

This section is organized as follows: (1) CNN-based FMD; (2) Hybrid FMD; (3) Hand-crafted FMD.

**Figure 4:**
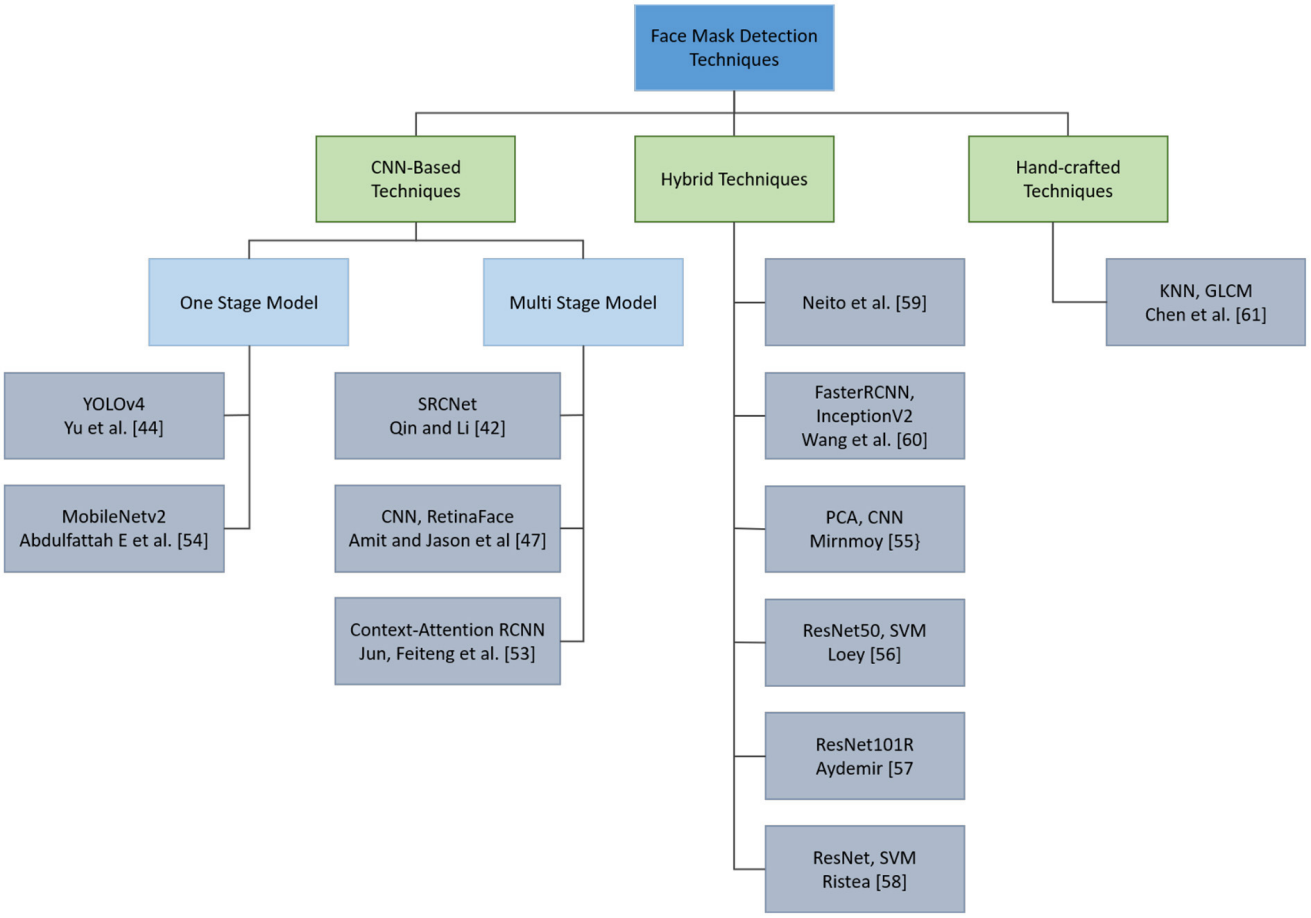
Hieratical representation of face mask detection techniques.

Figure 4 illustrates a hierarchical representation of the FMD techniques mentioned in this section.

#### CNN based FMD

This subsection covers works aiming at face mask detection using CNN algorithms. CNN has a remarkable feature extraction capability due to its pooling function and convolutional networks. ResNet, VGG- 16, MobileNet, DenseNet, and NASNet-Mobile are common CNN techniques that have been widely used for the task of face mask detection. The following techniques have been used:

Qin and Li (43) worked on developing a model that is capable of identifying the way a mask is worn. They developed a novel identification method named SRCNet which combines classification networks for classifying the mask arrangement and image super-resolution, which allows the model to recognize faces even with low resolution. The initial stage of the SRCNet network is the SR network, which was inspired by RED (44). CNN was employed for the second stage, which identifies the position of the face mask. The model has four major steps: image pre-processing for improving accuracy, facial detection and cropping to set the concentration only on the faces, image super-resolution that works on improving classification by upscaling blurred and low-quality faces, and finally face mask-wearing arrangement identification. It utilizes the public Medical Mask dataset for training and evaluation. The dataset has three classifications: no face mask wearing (NFW), incorrect face mask wearing (IFW), and correct face mask wearing (CFW). The model achieved a mean average precision of 98.7%.

To tackle the problem of high computational time and low-accuracy models, Yu et al. (45) introduced a Yolov4 model that uses an improved CSPDarkNet53 backbone. The neck of the model is composed of SPPNet and improved PANet. The CSPDarkNet53's rapid convergence can help to reduce training time and improve the learning ability of the model. The CSP2_X module was added to the PANet structure to improve the task of feature extraction. The SPNet network can work on enhancing the network's feature fusion capabilities. Finally, the model used an adaptive image scaling algorithm to decrease reasoning time by eliminating insignificant and redundant information from images. Besides the outstanding 98.3% mAP and 54.57 FPS results that were achieved by this model, it is an example of an effective solution for high complexity and training cost.

Tomás et al. (46) used CNN and transfer learning to detect and identify cases of mask misuse. The system detects different kinds of improper mask wear that are not taken into account in other studies, like mask adjusted below the bridge of the nose, glasses worn under the mask, incorrect placement of side rubber bands and more. The dataset limitation led the researchers to go and collect their own dataset. The dataset was labeled with the help of health professionals from Ontinyent hospital. For facial detection, Rapid Object Detection Using a Boosted Cascade (47) was employed. The system used the Adam technique as an optimizer. The model is available in the form of an Android app that can be found in Google Play (48). When using the app, faces on the frame will be detected. If there is no mask or the mask is worn improperly, a voice message will point that out. If the mask is being worn correctly, the person will be thanked for their adherence. Researchers decided to implement the model using MobileNetv2 as it is a small and fast model. The model achieved an accuracy of 0.812.

Chavda et al. (49) developed a two-stage CNN model that is compatible with CCTV cameras for detecting the way a face mask is worn. The first stage detects human faces, while the second stage localizes and classifies faces detected by the first stage as “MASK” or “NO MASK”. The task of the first stage is to detect multiple faces from different angles in an image. RetinaFace (48) was employed as the first stage model. RetinaFace is trained on a big dataset, which gives fast generalization and robust detection. The second stage CNN classifier was trained on three different models; namely, DenseNet121 (50), MobileNetV2 (51), and NASNet (52). Such models are known for their good performance and low latency, which makes them a good choice for real-time video analysis. Researches used an online scraping method for dataset collection. The created dataset contains 3,440 images of masked faces, and 4,415 images of faces with no masks. Densenet121 outperformed MobileNetV2, and NASNet with a precision of 99.7.

Context-Attention R-CNN is a new detection framework for wearing a face mask that enlarges intra-class distance and shortens inter-class distance by extracting distinguishing features, according to research published by Zhang et al. (53). For region proposals, first, a large number of context features are extracted, then the attention module is used to weigh these context features at the channel and spatial levels. The classification and localization branches are then segregated to acquire features that are more appropriate for these two tasks. Experiments show that the Context-Attention R-CNN achieves 84.1 percent mAP on the proposed dataset, outperforming Faster R-CNN by 6.8 points. The proposed dataset contains 8,635 faces in various states of wear and covers a wide range of scenarios.

Ba Alawi et al. (54) presented an automated recognition system for detecting individuals who violate face mask wearing procedures. Using deep learning techniques such as TensorFlow and Keras, this model recognizes masked faces automatically. This work efficiently distinguishes between masked and unmasked faces, allowing governments, businesses, and organizations to monitor and detect any violations of mask-wearing rules. Three pre-trained models were implemented in this work; namely, MobilenetV2, NASNetMobile, and DenseNet. MobilenetV2 had an accuracy of 0.9859, while NASNetMobile and DenseNet had 0.9758 and 0.9852, respectively. The purpose of this paper is to demonstrate the feasibility of recognizing masked faces effectively using a lightweight model that can be deployed even on low-resource platforms (e.g., mobile devices).

Table 4 shows a summary of works done on deep learning to detect face masks using CNN-based FMD.

**Table 4:** Summary of works performed using CNN based FMD.

**Reference**	**Methodology**	**Dataset**	**Performance**
Qin and Li (43)	SRCNet CNN	NFW IFW CFW	98.7% mean average precision
Yu et al. (45)	Yolov4 model	-	98.3% mAP and 54.57 FPS
Tomás et al. (46)	CNN Transfer learning MobileNetv2	Self gathered	81.2% Accuracy
Chavda et al. (49)	RetinaFace DenseNet121 MobileNetV2 NASNet	Online scraping method	Densenet121 got the best precision of 99.7
Zhang et al. (53)	Context-Attention R-CNN	-	84.1% mAP
Ba Alawi and Qasem (54)	MobilenetV2 NASNetMobile DenseNets	-	MobilenetV2 had 98.59% accuracy NASNetMobile had 97.58% accuracy DenseNets had 98.52 accuracy

#### Hybrid FMD

This section discusses studies undertaken for face mask detection using Hybrid Feature, an combination of deep learning, customized feature extraction and classical ML classifiers.

Bhattacharya (55) developed a face mask detector called HybridFace maskNet, which is a combination of classical ML and DL algorithms. On public faces, HybridFace maskNet can attain state-of-the-art accuracy. The suggested method employs a hybrid feature vector, which combines features from CNN-based deep learning methods with those extracted by a handcrafted feature extractor. Low-quality images, variable distances of individuals from the camera, and dynamic lighting on the faces in daylight or artificial light are the major obstacles. Hand crafted feature extractors are best for detecting features in low-quality images, whereas high-quality images are best for deep learning. As a result, classification accuracy can be improved by combining these two methods. HybridFace maskNet is trained on images with three different classifications: “proper-mask,” “incorrect-mask,” and “no-mask.” Images and real-time video streams were used to test the models. Though the system found the test accuracy to be around 62%, this accuracy could be upgraded.

Loey et al. (56) devised a face mask detection hybrid model based on both ML and DL-based approaches was proposed. To classify photos into masked and unmasked, the Resnet50 technique was used to extract features, which were then used to train the SVM, ensemble algorithm, and decision tree. In addition, three face-masked datasets have been chosen for analysis. The Real-World Masked Face Dataset (RMFD), the Simulated Masked Face Dataset (SMFD), and the Labeled Faces in the Wild (LFW) are the three datasets used for the study. In RMFD, the SVM classifier has a testing accuracy of 99.64%. It scored 99.49% testing accuracy in SMFD and 100% testing accuracy in LFW.

In this study by Aydemir et al. (57), Mask, no mask, and inappropriate masks were used to classify the individual photos. The following three cases were constructed based on these labels: Case 1 pits a mask against no mask versus an incorrect mask; Case 2 pits a mask against no mask + an improper mask; and Case 3 pits a mask against no mask. A hybrid deep-feature-based masked face classification model was trained and tested using this data. Pre-trained ResNet101 and DenseNet201 were employed as feature generators. In Case 1, Case 2, and Case 3, the resulting model achieved classification accuracy rates of 95.95, 97.49, and 100.0%, respectively. The suggested model is suitable for a practical experiment to detect appropriate face mask use in real-time, as seen by these high accuracy values.

Ristea and Ionescu (58) devised a method for detecting face masks from speech. This model had two parts: (1) using cycle-consistent GANs to transform unpaired utterances into two classes (with mask and without mask), and (2) using cycle-consistent GANs to assign opposite labels to each changed pronunciation, resulting in new training accents. The original and altered accents were converted to spectra, which were then fed into ResNet networks of various depths. The SVMs were classified to group the networks. The model was trained using augmented spectrograms in this procedure. G and G' were also used to convert training spectrograms from one class to another. The data augmentation method used in this work outperformed other baseline techniques. However, the model requires a long processing time. As a result, the fundamental flaw in this approach is the ratio of time consumption to accuracy. Researchers have examined their outcomes with and without data augmentation and found that the augmented data has a performance boost of 0.9%, achieving 74.6% accuracy.

Nieto-Rodríguez et al. (59) introduced a real-time detection system for face masks that sounds an alarm when medical or operating room personnel are not wearing surgical masks. For each detection, they employed two detectors and two-color filters. A face detector was one of them, and a medical mask detector was another. The classic Viola-Jones face detection technique was used to detect faces. On 496 faces and 181 masks from the BAO database, as well as an original image dataset for faces with masks, the positive and false positive rates are above 95 percent and below 5%, respectively. On a standard PC, the system operates in real time.

Wang et al. (41) introduced a two-stage process to detect the application of masks using hybrid machine learning algorithms. A user wearing a face mask is detected in the first stage, utilizing the Faster RCNN and InceptionV2 structural model. The second phase leads to a stage where real face masks generated with a learning system are verified by a classifier using a learning system. In addition, this research presents a data set of 7,804 realistic pictures for detecting faces wearing masks (WMD). There are 26,403 masks in the data set, which covers a wide range of scenarios. The overall accuracy for basic scenarios is 97.32 percent, whereas it is 91.13 percent for more difficult scenarios. Table 5 shows a summary of the works carried out on deep learning to detect face masks using Hybrid FMD.

**Table 5:** Summary of works carried out using Hybrid FMD.

**Reference**	**Methodology**	**Dataset**	**Performance**
Bhattacharya (55)	HybridFace maskNet		62% accuracy
Loey et al. (56)	Resnet50 SVM Ensemble algorithm Decision tree	Real-World Masked Face Dataset (RMFD) Simulated Masked Face Dataset (SMFD) Labeled Faces in the Wild (LFW)	SVM 99.64% had accuracy on RMFD 99.49% accuracy on SMFD and 100% accuracy on LFW
Aydemir et al. (57)	Pre-trained ResNet101 and DenseNet201		95.95, 97.49, and 100.0% accuracies for the three cases.
Ristea and Ionescu (58)	ResNet SVM		74.6% accuracy
Nieto-Rodríguez et al. (59)	Viola-Jones face detection technique	BAO database	
Wang et al. (41)	RCNN and InceptionV2	WMD	97.32% accuracy for basic scenarios 91.13% accuracy for more difficult scenarios

#### Hand-crafted FMD

Hand-crafted methods are considered traditional object detection approaches. Finding proposals, extracting features, and categorization are all common phases in these approaches. The majority of successful classical object detection algorithms relied on carefully designing feature descriptors to acquire embedding for a region of interest. Haar, SIFT, SURF, and HOG are some examples of hand-crafted techniques. The number of studies that used only hand-crafted methods is limited, as they are usually combined with deep learning-based techniques.

Chen et al. (60) proposed a mobile phone-based detection system. We start by extracting four features from the GLCMs of micro-photos of the face mask. A three-result detection system is then created using the KNN algorithm. On the testing dataset, the results of validation trials demonstrate that our system can achieve a precision of 82.87 ± 8.50%.

**Figure 5:**
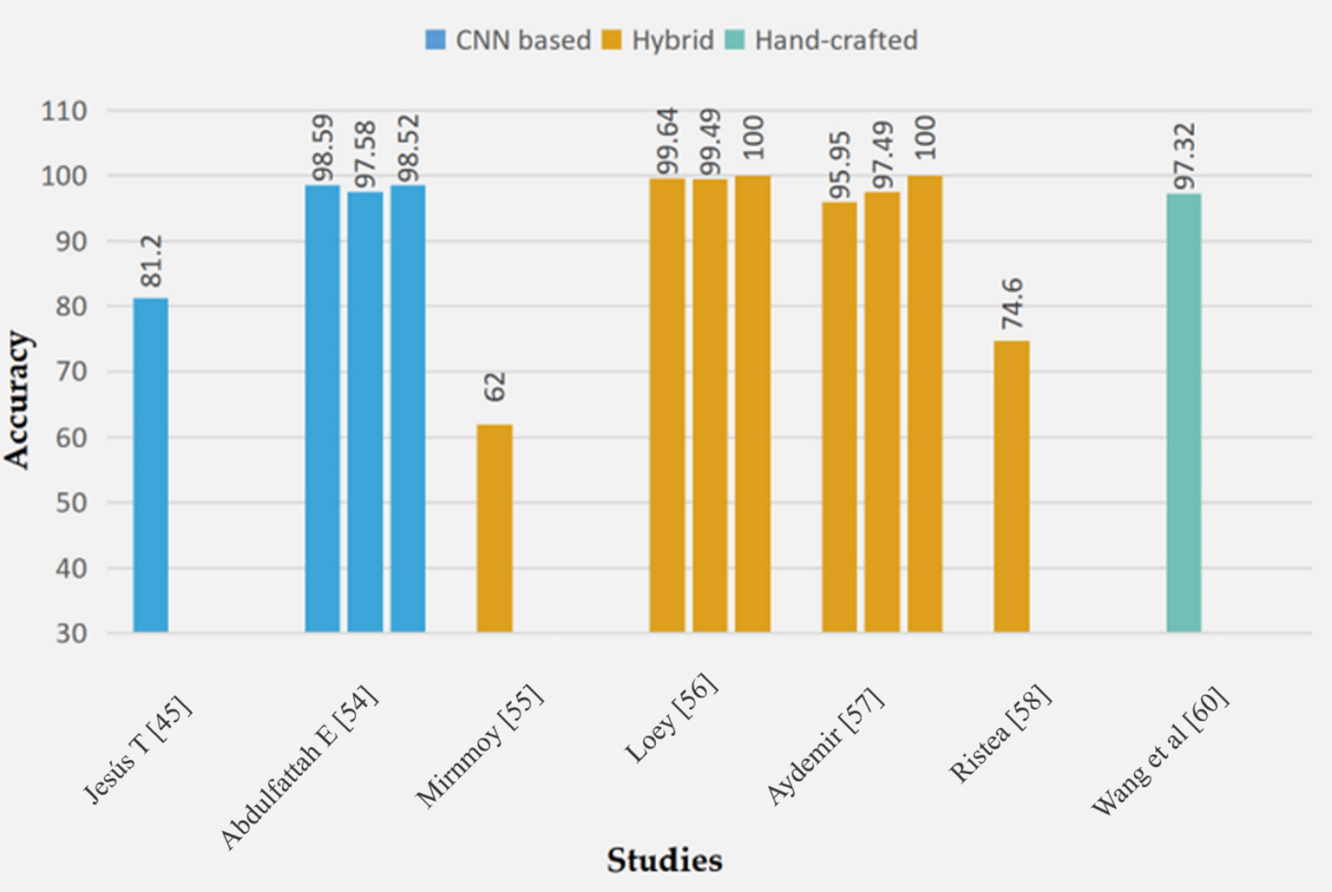
Performance accuracy measures of different FMD models.

In this section, we reviewed the efforts made in the field of face mask detection. The studies carried out are sub-divided into three (CNN, hybrid and hand-crafted). A graphical representation of the performance of these techniques is seen in Figure 5.

## State of the art models

**Figure 6:**
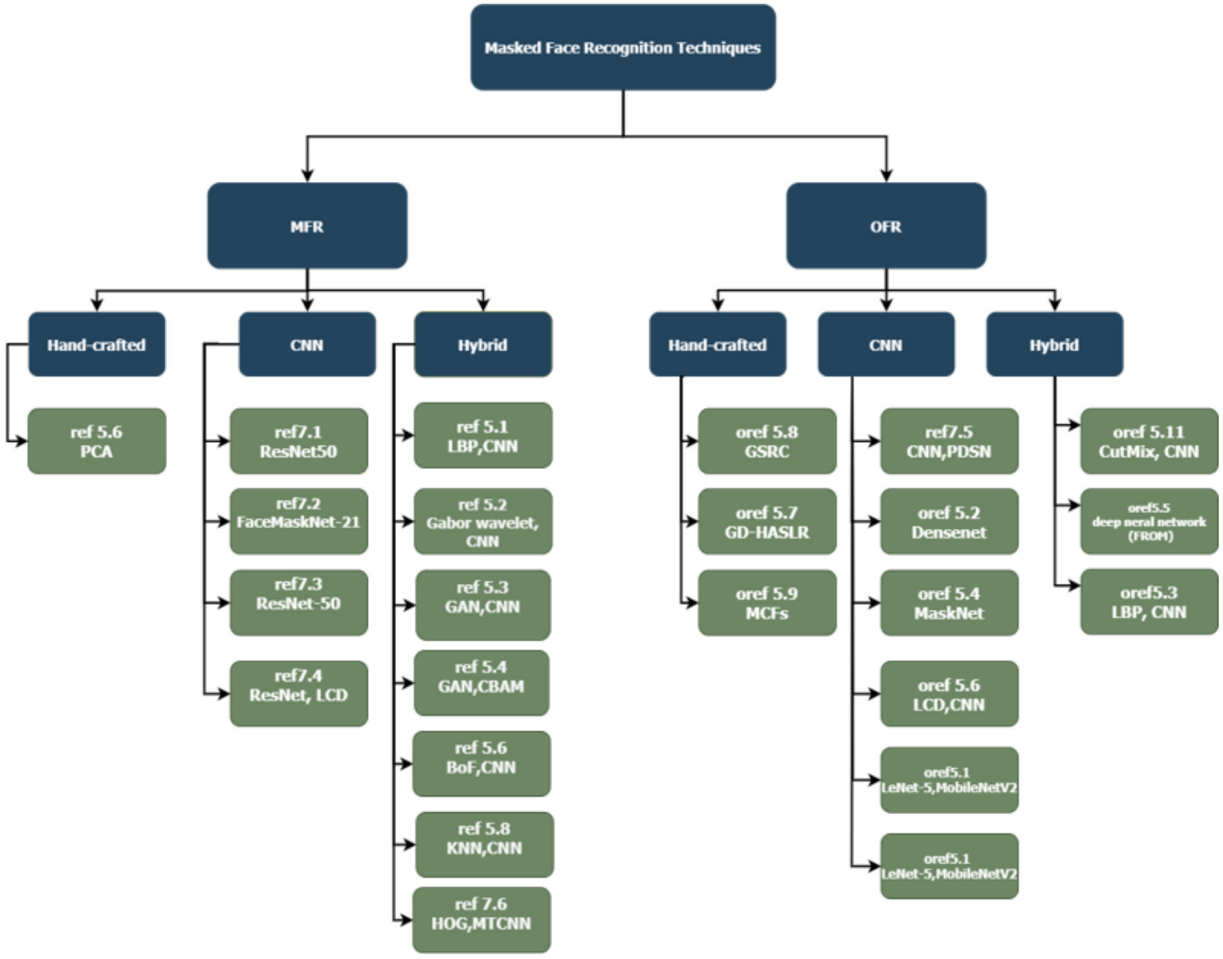
Hieratical representation of masked face recognition techniques.

This section reviews methods used to recognize masked faces, which can be split into two approaches: MFR, studies and methods that were developed specifically for the MFR problem; and OFR, studies that cover different types of occluded face recognition problems, including recognizing faces covered with a scarf. These studies are further divided based on the technique they used (Hand-crafted, CNN, Hybrid). A hierarchical representation of current state-of-the-art of algorithms covered in Section Challenges is illustrated in Figure 6.

### MFR

**Figure 7:**
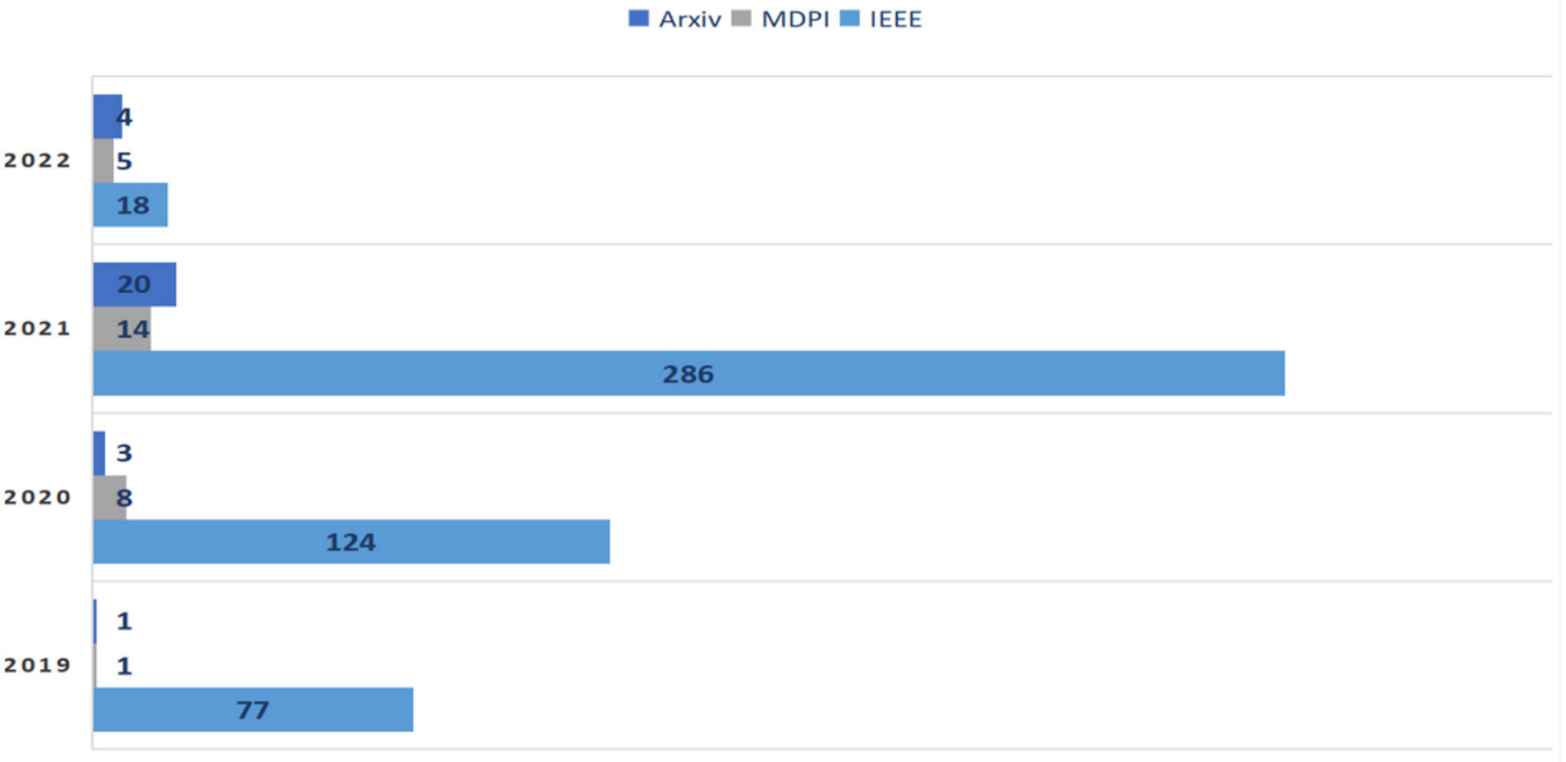
Demonstration of research efforts on face mask detection from 2019 to 2022.

During the COVID-19 pandemic, people used face masks more than ever before, which explains the dramatic increase in published MFR studies during the pandemic, as the demand for MFR solutions increased with the rise in face mask usage. A search query was performed on the biggest online libraries to track the effort on MFR over the years. Figure 7 clearly indicates that the increase in research related to MFR goes hand-in-hand with the spread and increase of COVID-19.

Vu et al. (61) extracted features such as eyebrows and eyes from masked input photos, using a mix of CNN and LocalBinary Pattern (LBP). The system outperforms state-of-the-art models in terms of efficiency. However, the created technology cannot be used on portable embedded systems due to its relatively high energy consumption. Researchers working on this study developed a dataset, named COMASK20, which contains 2,754 labeled images from 300 different individuals. On the COMASK20 dataset, the recognition rate was 87% f1-score, and on Essex, it was 98% f1-score.

Durga et al. (62) combined Gabor wavelet and deep transfer learning to solve the problem of face and veil identification. The Gabor wavelet features are recovered from the non-masked part of the face and combined with deep learning CNN features to create a more robust feature vector that can be used to improve recognition. The suggested method was tested on four benchmark datasets and a manually produced dataset, achieving an average recognition accuracy of 97%.

Golwalkar et al. (63) proposed a solution that uses deep metric learning and their own FacemaskNet-21 deep learning network to build 128-d encodings for face recognition in static pictures, live video streams, and static video files. With an execution time of less than 10 milliseconds, they were able to obtain a testing accuracy of 88.92%.

In the study by Montero et al. (64), the problem of face recognition with masks is addressed. Based on the ArcFace work, a complete training pipeline is developed, with many changes to the backbone and loss function. Using data augmentation, a masked version of the original face-recognition dataset is created, and the two datasets are mixed during the training phase. The chosen network, which is based on ResNet-50, is modified to assess the probability of mask usage without incurring any additional computational costs. Experiments show that the proposed method greatly improves the accuracy of the original model when dealing with masked faces, while maintaining nearly the same accuracy on non-masked datasets. In addition, it has a mask-usage categorization accuracy of 99.78 %.

Li et al. (65) combined a cropping-based strategy with the Convolutional Block Attention Module, a new technique for masked face identification was proposed (CBAM). The best cropping for each example is investigated, and the CBAM module is tweaked to focus on the areas around the eyes. Extensive tests on the SMFRD, CISIA-Web face, AR, and Extend Yela B datasets show that the proposed approach can greatly enhance masked face recognition performance when compared to other state-of-the-art approaches.

Ejaz et al. (66) offered an automated Masked Face Recognition (MFR) system using a deep-learning model and a mask occlusion rejecting technique. The photos are first passed through three filters in a pre-processing step. Then, to extract characteristics from non-occluded portions of the faces (i.e., eyes and forehead), a Convolutional Neural Network (CNN) model is presented. Covariance-based features are obtained using these feature maps. Based on the Bag-of-Features (BoF) paradigm, the features of the deep covariance are quantized to codebooks. Bitmap and Eigenvalue are two additional layers that are used to lower the dimension and concatenate these covariance feature matrices. The system achieves 95.07 percent and 92.32 percent accuracy, respectively, demonstrating its competitive performance when compared to the state of the art.

The Deep Convolutional Neural Network (CNN) model and machine learning classifiers for masked face recognition are evaluated in the study by Razali et al. (67). This study specifically examines the feature extractors DENSENET201, NASNETLARGE, INCEPTIONRESNETV2 and EFFICIENTNET (EFFNET). The collected characteristics are then categorized using Support Vector Machines (SVM), Linear Support Vector Machines (LSVM), Decision Trees (DT), K-nearest Neighbor (KNN), and Convolutional Neural Networks (CNN). The face mask detection dataset is used to test the recognition model. The results of the trial revealed that DENSENET201-SVM and EFFNET-LSVM had the highest classification accuracy of 0.9972. EFFNET-LSVM, on the other hand, has a faster computing time for feature extraction, classification, and feature size. Table 6 shows a summary of these MFR-based models.

**Table 6:** Summary of works using MFR.

**Reference**	**Methodology**	**Dataset**	**Performance**
Vu et al. (61)	CNN LocalBinary Pattern (LBP)	COMASK20 Essex dataset	87% f1-score on COMASK20 98% f1-score on Essex
Durga et al. (62)	Gabor wavelet CNN	Self-gathered	97% average recognition accuracy.
Golwalkar and Mehendale (63)	Face maskNet-21		88.92 % testing accuracy
Montero et al. (64)	ResNet-50		99.78 % categorization accuracy
Li et al. (65)	CBAM	SMFRD CISIA-Web face AR Extend Yela B datasets	Very high performance
Ejaz et al. (66)	Masked Face Recognition (MFR) CNN		95.07% accuracy for MFR 92.32% accuracy for CNN
Razali et al. (67)	EFFNET LSVM	Face mask detection dataset	99.72% accuracy

### OFR

Unlike MFR, OFR is independent of COVID-19. It is seen as one of the key challenges in FR. Here, we review some of the efforts made in the field of OFR, particularly OFR studies covering face mask-like situations, which are therefore applicable to MFR.

For face recognition on mask-occluded face photos, Raihan and Santoni (68) used two convolutional neural network (CNN) architectures: LeNet-5 and MobileNetV2. Cropping, artificial mask augmentation, scaling, and image augmentation were used to preprocess data from 12 people who were photographed face to face. The model was trained for 50 epochs with a 60:40 data split using the set hyperparameter. Model testing was carried out on picture data without the use of augmentation or a mask. The classification accuracy of the test findings was tested for 12 classes. Testing accuracy on LeNet-5 models was 98.15%, while MobileNetV2 testing accuracy was 97.22%.

When the entire face is not visible, occluded face identification becomes significantly more difficult. Jiayu (69) observed that the problem has not been fully resolved and that it has remained a hot topic in recent years. In this study, the real-world masked face dataset was evaluated on five advanced models with the goal of comparing their performance. Among these 5 different models, DenseNet performed the best, with a test accuracy of 0.8012.

Wan and Chen (70) proposed MaskNet, a trainable module that can be incorporated into existing CNN architectures. MaskNet learns an appropriate approach of adaptively creating distinct feature map masks for diverse occluded face photos with end-to-end training supervised exclusively by personal identification labels. MaskNet assigns larger weights to hidden units activated by non-occluded facial parts and lower weights to those activated by occluded facial parts based on intuition. Experiments on real-life and synthetic occluded face datasets show that MaskNet may effectively improve the resilience of CNN models in face recognition when faced with occlusions.

Another study by Qui et al. (71) proposed a novel face recognition algorithm that is robust against occlusions. FROM (Face Recognition with Occlusion Masks) is a method for learning to detect corrupted features in deep convolutional neural networks and cleaning them with dynamically learned masks. A huge dataset of occluded face pictures was also created, to effectively and efficiently train FROM. When compared to existing techniques that either rely on external detectors to detect occlusions or use shallow models that are less discriminative, FROM is simple yet powerful. The LFW, Megaface Challenge 1, RMF2, AR dataset, and other simulated occluded/masked datasets all show that FROM improves accuracy significantly under occlusions.

A novel and elegant occlusion-simulation method is proposed by He et al. (72), based on the idea that occlusion basically hurts a group of neurons, by lowering the activation of a group of neurons in some meticulously selected channel. A spatial regularization technique is used first to encourage each feature channel to adapt to local and diverse face areas. Then, by dropping out a few feature channels, the locality-aware channel-wise dropout (LCD) is used to simulate occlusions. The suggested LCD can encourage subsequent layers to reduce intra-class feature variance caused by occlusions, resulting in increased occlusion robustness.

In a paper by Wu and Ding (73), the continuous face occlusion recognition problem is solved using a very effective method. It employs a hierarchical sparse and low-rank regression model, as well as robust image gradient direction features and a range of mapping functions. This approach combines the sparse representation of dictionary learning with the low-rank representation of the error term in the gradient domain, which is typically messy. It is known as the “weak low-rankness” optimization problem, and is easily addressed using the Alternating Direction Method of Multipliers framework (ADMM). The proposed gradient direction-based hierarchical adaptive sparse and low-rank (GD-HASLR) algorithm outperforms state-of-the-art methods, including prominent convolutional neural network-based methods, in these studies.

Borges et al. (74) worked on recognizing faces with occlusion using a robust classifier based on a ResNet backbone. This study covers 8 types of face occlusion, including the occlusion caused by a face mask. A subset of 5,478 images from the CelebA-HQ was used for training the classifier. The 8 types of face occlusion were added to the input images, which cause a huge decrease in the classifier performance. To enhance the performance under occlusion, researchers used two approaches. The first is image inpainting, which is reconstructing the missing part of an image. Image inpainting is done using the pre-trained broad-based image completion network. The second approach is Cutmix, which aims to make the classifier less prone to information loss and input corruption. Both approaches showed good results.

Image Gabor-features are employed for SRC in the work done by Yang and Zhang (75). The occlusion dictionary is compressed using Gabor kernels, and a Gabor occlusion dictionary calculation algorithm is subsequently provided. The number of atoms is significantly reduced in the computed form. The Gabor occlusion dictionary minimizes the computing cost of coding occluded face photos while also enhancing SRC accuracy. The suggested Gabor-feature based SRC (GSRC) scheme was proved to be effective in experiments on representative face databases with differences in lighting, expression, position, and occlusion.

Correlation Filters (CFs) are an occlusion-tolerant object recognition approach that can handle partial occlusions. Correlation Filters (CFs) are a partial occlusion-tolerant object recognition technique. As reported by He et al. (76), researchers develop Masked Correlation Filters (MCFs), a novel class of correlation filters built specifically to deal with partial occlusions in facial images. The benefits of using MCFs are illustrated using well-known face image data sets.

Zeng et al. (77) restricted their scope to occluded face recognition. The first thing they did was explore what the occlusion problem was and how difficulties arose. Then they presented how existing face detection algorithms could tackle the problem of occlusion and classified them into three categories: (1) occlusion-robust feature extraction approaches, (2) occlusion-aware face recognition approaches, and (3) occlusion-recovery-based face recognition approaches. Furthermore, they analyzed the motivations, innovations, pros and cons, and the performance of representative approaches for comparison of the existing methods. Finally, future challenges and method trends of occluded face recognition were thoroughly discussed and analyzed.

### Pipeline

**Figure 8:**
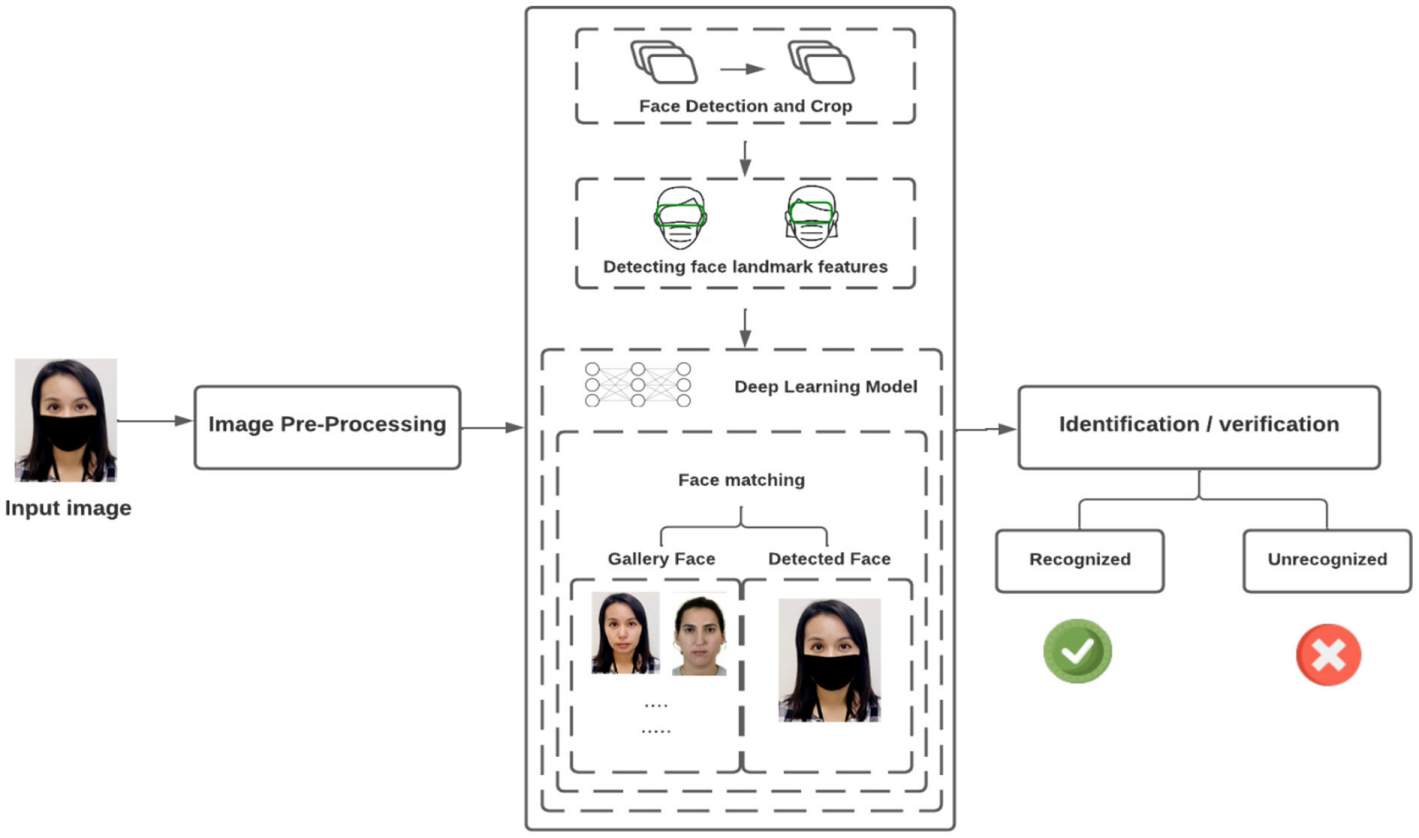
Pipeline for face mask detection.

This section explains how MFR systems are usually created. To discover the major aspects of the face, we first use the Deep Learning Model. As shown in Figure 8, there are a number of processes in constructing this system, which we will go over in the subsections below.

#### Image preprocessing

Image pre-processing is used to increase the efficiency of models trained using the dataset because it is a form of cleaning. Because raw photographs captured in real life have extensive variation in contrast and exposure, image pre-processing is required to ensure that facial detection and face-mask-wearing status identification are accurate (43). There are only a few publicly available datasets. As a result, it is even more important to add synthetic photographs with various types of face masks to the testbed, as well as improve the generalization capabilities of deep learning models. The data pre-processing stages are carried out to clean the available data and remove elements which will hinder the performance, such as smoke and images that are corrupted, blurred or missing important parts.

#### Data augmentation

Data Augmentation is a widely used approach for getting the most out of a data source. To increase the variety of the training set, tiny changes to the images are made, such as slight rotations, translations, and zooming in the input images (46). Data augmentation can improve the performance and results of machine learning models by producing new and varied cases to train datasets. Due to the fact that machine learning algorithms require a large dataset to train effectively and available datasets are scarce, especially for relatively newer fields of research, data augmentation is required to increase the available range of datasets by making alterations to those already available.

#### The initial stage is face detection and cropping

Thanks to progress in deep-learning and convolutional neural networks (CNNs), face detection models have shown a huge improvement over the past decade. Consequently, the majority of existing state-of-the-art face-detection methods are actually based on convolutional neural networks and are capable of effectively detecting faces with difficult characteristics and with differences regarding scale, posture and lighting, from datasets that are low in quality and in the company of various obstructing elements. Here are some of the outstanding models:

- The Dual Shot Face Detector (DSFD) (13):- extending the single shot detector to dual shot detection was achieved by using a Feature Enhance Module (FEM) to enhance the original feature maps. The Dual Shot Face Detector has some improvements over SSD in regard to descriptive feature maps, a better learning objective (loss), and an upgraded method of matching predictions to the faces in the input images.^1^
- Multi-Task Cascaded Convolutional Neural Network (MTCNN) (14)—MTCNN is a thoroughly made cascaded CNN-based model for both face detection and alignment. The MTCNN's process of detection has three stages of convolutional networks, making it capable of predicting faces and landmark locations in a coarse-to-fine manner.^2^
- The AIZooTech mask detector —The AIZoo face mask detector is an open-source detector that utilizes the structure of SSD (15). To guarantee low computational complexity, it employs a lightweight backbone network.
- RetinaFace (16)—RetinaFace is a single-shot multi-level face localization model which applies pixel-wise face localization on different measures by taking advantage of multi-task learning. It collectively conducts three distinct face localization tasks, i.e., 2D face alignment, face detection, and 3D face reconstruction within a single model.^3^

To improve the accuracy of any face detector, it has to focus on information from the face itself, rather than the background or any other distractors, especially since some images have differences in expression, face size, age bracket and background. Hence, a robust and highly accurate face detector is needed. To achieve that, a cropping technique could be used. It works by cropping faces from the pre-processed image to serve as inputs after locating their positions.

#### Feature extraction

Before processing techniques such as face tracking, facial expression detection, masked face recognition, and face recognition can begin, features from the face must be extracted. Currently, images of human faces are used to extract features such as eyes, noses, mouths, and other facial features. However, this is becoming more difficult with COVID-19 because the face mask covers half of the face. Through the conversion process, the image is converted into data based on a person's facial features, which allows the system to identify the most relevant information from the images without taking into account any noise. Feature extractions in facial recognition may result in packing of information, noise reduction, dimension reduction, and salience extraction. Each person's faceprint is extracted during the feature extraction process.

**Figure 9:**
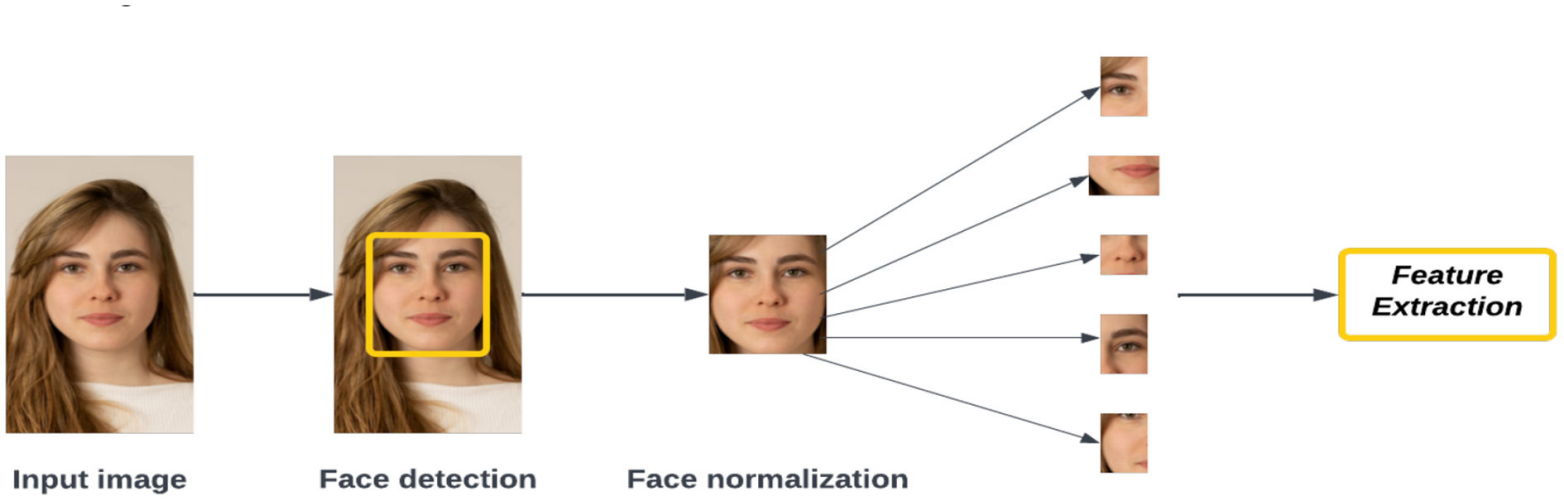
Steps to feature extraction.

Feature extraction approaches for masked face recognition are categorized into shallow and deep representations. Deep feature extraction uses hand-crafted features without requiring much learning or optimization. It is a traditional method of hand-crafting features with limited learning and optimization mechanisms. The occluded local parts can be found using handcrafted low-level features and they are excluded from the recognition process. Holistic learning approaches, local features, and shallow learning approaches are all described by LBPs (63), PCAs (66), and HOGs (78). Face recognition has been achieved using non-occluded tasks that are robust and accurate against many face changes, such as illumination, affine, rotation, scale, and translation. Deep-learning models have been able to significantly outperform shallow features when dealing with occluded faces, including masks, because deep representations have been obtained through deep learning. In Figure 9, we show the most significant features found in an extracted image.

#### Face matching and recognition

A final step in the system is to identify the identities of the faces of the individuals. For an automated recognition system, a face database can be created by taking several images of each individual's face, then analyzing the features of these images and storing them in the system database. When an input image is presented, the system will perform face detection and feature extraction on the image. As a result, the system compares the image features against each faceprint in the database and grants or denies access. Identification and verification are two different applications of facial recognition. The system is requested to tell who the given image is for when it comes to facial recognition. Face verification, on the other hand, requires the system to determine whether an identified image is true or fraudulent (79).

#### Face restoration

Face restoration is the process of “filling” the missing part of the face. In the case of MFR, the occluded missing part is the mask area. The first step in face restoration is to generate a binary segmentation map of the detected mask region. The second step is to pass the segmentation map as well as the input image (i.e., the masked face image) into a network to remove the mask object and fill the affected area. For this task, a GAN based-network is used due to their strong learning ability. It is noteworthy that non-learning-based MFR algorithms are restricted to the removal of small objects only, whereas learning-based algorithms, GLCM for instance, complete the random affected area in facial images.

The challenge here is not only to generate visually plausible faces, but also to represent accurate resemblances of the real face. Many MFRs do not use face restoration because of the algorithm's limited ability to effectively handle mask removal and restoration. Yet, there are some strong published studies that utilize face restoration in the domain of MFR (80–82). Face restoration in the domain of MFR is uncommon, however, since the algorithm's accuracy in handling mask removal and restoration is doubtful.

## Challenges

This section draws attention to the major challenges, difficulties, and issues surrounding the work covered in this survey.

### The issues of available datasets

Here, we address the issues related to face mask datasets.

- Binary classification datasets.

For face mask detection, the task should not be limited to detecting the mask and ignoring the way it is worn. An improperly worn mask has the same health risks as wearing no mask. Identifying individuals with improper face masks in the same category as individuals with the correct masks is, therefore, invalid and those individuals will continue their bad habits. Also, when employing these datasets in models to analyze people's adherence to the wearing of face masks, the results will be misleading.

- Barely balanced datasets.

Having one class with more instances than the others make the model biased toward predicting the class of higher instances, and in the case of a class that is severely underrepresented, the model might ignore that class completely. The result is a model that does not perform properly.

- Unclassified mask type issue.

The varying types of masks entail varying levels of protection. A mask of type N95 provides higher protection than a regular medical mask, which also differs from a cloth or satin mask. Almost all the face mask datasets fail to categorize mask types. A dataset that provides a categorization of face mask types is needed. This is especially important for models that serve in domains where the mask type matters, such as industrial locations that involve working with chemicals, or medical laboratories. Also, feeding the dataset with different types of masks makes it more robust, particularly in detecting fashionable commercial masks that come in the color of the skin.

- Unvarying faces with the same orientation.

A dataset that has face images from only one angle is not enough for proper detection. During the work on this survey and whilst reviewing the existing datasets, we noted that most datasets contain frontal face images and lack profile images. Moreover, most of the faces belong to the same age group and same race. Black people, Asian people, children, and elderly people were not included in most available datasets. In the absence of these features, a dataset cannot cover all real-life scenarios, and therefore cannot provide reliable results.

### The challenge of recognizing masked faces

Masked face recognition is an extreme case of occluded face recognition, since almost the whole face is covered. MFR has gained attention only recently, due to the face mask-wearing policy imposed during COVID-19, which may explain the limited range of studies. Most MFR systems employ the techniques of OFR instead of developing a framework that can handle the MFR from all angles. The existence of robust MFR models could serve different applications. An excellent MFR framework could boost security systems and hence reduce crime rates. The use of MFR for authentication can eliminate the need to remove masks for face matching, thereby reducing the risk of contagious diseases spreading.

### The computational complexity of models

The model's computational complexity reflects its feasibility for deployment in real-time systems and CCTVs. A practical and feasible system is a system that is fast, accurate, light-weight, and does not consume excessive memory. However, it is extremely difficult to maintain robust performance with lightweight, fast models for face mask detection. Despite the importance of computational complexity, we found that most papers did not mention how well their models performed in this respect.

## Research future and conclusion

In this study, we reviewed the main scope and contributions of AI in relation to COVID-19. The latest trends in the field of face mask detection, as well as masked face recognition, were surveyed. In this work, more than 30 papers were reviewed for both FMD and MFR. A taxonomy is presented that summarizes the approaches of both fields. This study also discusses and reviews 13 of the largest publicly available face mask datasets. Finally, we addressed the main challenges and difficulties related to the topics covered in this survey. During this work, we observed that most of the MFR techniques are actually based on pre-existing FR techniques, which explains the unsatisfactory performance of the MFR. Moreover, it is noted that the majority of available datasets are lacking in some respects; the optimal dataset is rarely found. Also, there is a need for comparative analysis of deep learning models in terms of their processing speed and computational performance. In the end, we hope that this survey provides useful research recommendations that can serve as an inspiration for future studies.

## Author contributions

All authors listed have made a substantial, direct, and intellectual contribution to the work and approved it for publication.

## Conflict of interest

The authors declare that the research was conducted in the absence of any commercial or financial relationships that could be construed as a potential conflict of interest.

## Publisher's note

All claims expressed in this article are solely those of the authors and do not necessarily represent those of their affiliated organizations, or those of the publisher, the editors and the reviewers. Any product that may be evaluated in this article, or claim that may be made by its manufacturer, is not guaranteed or endorsed by the publisher.

## Footnotes

^1^ https://github.com/hukkelas/DSFD-PytorchInference

^2^ https://github.com/matlab-deep-learning/mtcnn-face-detection/releases/tag/v1.2.3

^3^ https://github.com/biubug6/Pytorch_Retinaface
